# Defining and measuring diet quality across populations in nutritional epidemiology

**DOI:** 10.1017/S1368980025101559

**Published:** 2025-12-12

**Authors:** Kath Roberts

**Affiliations:** Hull York Medical School, University of York, Heslington, York YO10 5DD, UK

**Keywords:** Diet quality, Dietary assessment, Dietary patterns, Dietary surveillance

Assessing the quality of diets at the population level has become a cornerstone of public health nutrition research, offering a comprehensive lens through which to evaluate habitual dietary intake and its associations with health outcomes. In recent decades, ‘diet quality’ has emerged as a powerful construct in nutritional epidemiology that attempts to capture the multifaceted characteristics of habitual dietary patterns. Yet, defining and measuring diet quality is fraught with conceptual, methodological and contextual challenges. Variability in national dietary guidelines, cultural differences in food patterns and methodological and practical limitations in dietary data collection make it difficult to measure diet quality in a consistent, meaningful and scientifically rigorous way. These challenges limit our ability to compare findings across populations and time or to use diet quality indices as effective tools for surveillance, intervention or policy making.

## The conceptual complexity of ‘diet quality’

The term ‘diet quality’ encompasses several interrelated ideas – adequacy, balance, moderation and variety, each reflecting distinct but overlapping aspects of dietary intake^(1)^. Broadly speaking, diet quality reflects adherence to dietary patterns that are associated with improved health outcomes. These patterns typically include high consumption of fruits, vegetables, legumes, whole grains and healthy fats, with limited intake of free sugars, Na, red and processed meats, processed foods^(1)^ and more recently ‘ultra-processed’ foods^(2)^. Yet operationalising these seemingly intuitive and straight forward principles into measurable indicators or indices that are valid across populations is inherently complex. Definitions often reflect normative standards derived from national dietary guidelines, which themselves differ across countries and evolve over time^(2)^. The question of whether we should define diet quality according to national dietary guidelines^(3)^, international recommendations such as those from WHO or epidemiological evidence of disease risk is a difficult one. Each approach brings strengths and limitations.

## Tools used in high income countries and for global assessment of diet quality

In high-income countries such as the UK, the USA and Australia, diet quality scores have been developed around national guidelines. However, these fundamental guidelines vary in how they define healthy eating patterns and inevitably the associated constructs then vary in their building blocks. For instance, in the UK, the Eatwell Guide, primarily a public health messaging tool, emphasises food groups with minimal quantitative specificity and exists alongside more detailed dietary reference values and estimated average requirements for energy and nutrients (UK Scientific Advisory Committee on Nutrition, 2011^(4)^ 2015^(5)^, 2016^(6)^). US Dietary Guidelines incorporate numerical limits on nutrients such as added sugars and saturated fats^(7)^. Australia’s guidelines, meanwhile, stress food variety and discretionary food limits^(8)^.

National guidelines differ in emphasis, nutrient targets and food group categorisations. For example, the UK’s Eatwell Guide does not specify nutrient thresholds, unlike the US Dietary Guidelines, which underpin the Healthy Eating Index^(7)^. Australia’s guidelines stress food group variety and discretionary food limits^(8)^. This diversity makes cross-country comparisons difficult and raises critical questions about what constitutes a healthy diet across settings.

In the UK, the National Diet and Nutrition Survey offers one of the most detailed and biomarker-validated dietary datasets globally. Data from weighed food diaries, 24-h recalls and blood and urine samples allow researchers to develop both nutrient-based and food-based indices. Data from this cohort study have been used to derive empirically based dietary patterns in UK adults, which were then compared with nutrient biomarkers and socio-demographic variables and scored against a Nutrient-based Diet Quality Score based on UK DRV^(9)^. Building on this, the UK Diet Quality Questionnaire was developed as a brief food-based score^(10)^. While this tool is pragmatic and culturally relevant, its food-based format limits its ability to quantify nutrient-level adequacy or capture emerging issues such as ultra-processed food intake.

Europe presents a particularly diverse dietary landscape. The Mediterranean Diet Score is the most widely used index, especially in southern Europe. It assigns points for high intakes of plant foods, olive oil and fish, with penalties for red meat and dairy^(11)^. Meta-analyses show strong inverse associations between Mediterranean Diet Score scores and non-communicable disease outcomes, including CVD and cancer^(12)^. However, this tool is culturally specific; scoring poorly on the Mediterranean Diet Score may not indicate poor diet quality in non-Mediterranean settings, where oils and wine are less commonly consumed.

The WHO healthy diet indicator provides a nutrient-based index suitable for international comparisons. Based on WHO guidelines for chronic disease prevention, the Healthy Diet Indicator includes cut-offs for total fat, saturated fat, sugar, Na and fibre^(13)^. Despite its universality, the Healthy Diet Indicator is infrequently used in population surveillance due to its reliance on high-quality nutrient intake data.

To address cross-country heterogeneity, the European Prospective Investigation into Cancer and Nutrition study adopted a novel approach. European Prospective Investigation into Cancer and Nutrition collected dietary data from over 500 000 participants in ten countries using different local instruments. To enable valid comparisons, a calibration sub-study was conducted using 24-h recalls in a random subset of 36 900 participants. These data were used to correct for systematic measurement error and align dietary intake variables across countries^(14)^. This approach enabled pooled analyses and provided insights into regional differences in diet–disease relationships while highlighting the resource-intensiveness of calibration methods.

Nationally tailored tools such as the Dutch Healthy Diet Index (DHD15)^(15)^ the French PNNS-GS2^(16)^ and KidMed for Spanish children^(17)^ have also emerged. These hybrid indices combine food- and nutrient-based metrics but often lack cross-validation outside their source population.

The Healthy Eating Index remains the most widely used diet quality index in the US. Updated every five years to reflect new dietary guidelines, the Healthy Eating Index-2015 includes thirteen components across adequacy and moderation domains, scored proportionally to energy intake^(7)^. The Healthy Eating Index has been validated against both health outcomes and nutrient biomarkers and is considered a gold standard for large-scale epidemiologic studies^(18)^.

Derivatives such as the Alternative Healthy Eating Indexand the DASH score (based on the Dietary Approaches to Stop Hypertension trial) offer refinements targeting chronic disease prevention^(19,20)^. However, these indices require detailed dietary recalls and reflect Western dietary patterns, reducing their portability.

Australia’s tools emphasise pragmatic and food-based assessment. The Australian Recommended Food Score is a variety-based index built on a 70-item FFQ^(8)^. It focuses on frequency and diversity within core food groups but does not penalise for unhealthy food intake^(8)^. Other tools such as the Dietary Guideline Index^(21)^ and the Healthy Eating Index for Australians-2013^(22)^ incorporate both positive and negative scoring.

Another tool frequently applied in diet quality research is the Diet Quality Index (DQI)^(23)^. Unlike some other indices, the DQI is structured around the four domains of variety, adequacy, moderation and balance, providing a multidimensional framework for assessing diet quality. As other examples, it is a hybrid tool that combines both food-based elements (e.g. variety of food groups) and nutrient-based criteria (e.g. adequacy of fibre, Fe and Ca and vitamin C). By incorporating universal elements such as nutrient adequacy and moderation and retaining flexibility to assess culturally specific foods, the DQI-I has been applied in comparative studies, for example between China and the United States, demonstrating its utility as a harmonised yet adaptable measure of diet quality across diverse contexts^(24)^.

## Food-based v. nutrient-based tools: trade-offs

Food-based tools are more intuitive and feasible for large-scale or low-resource settings. They are closely aligned with public messaging and typically involve less participant burden. However, they lack granularity and may mask overconsumption of energy-dense foods. Nutrient-based indices offer precision and are sensitive to deficiencies or imbalances, but they are harder to measure and often ignore cultural eating patterns^(3)^.

As described above, most modern tools now blend both approaches. Hybrid indices such as the Healthy Eating Index-2020^(18)^, the DQI and the DHD15^(15)^ integrate food variety with nutrient thresholds, improving validity while maintaining feasibility. Nevertheless, harmonising these tools across cultures, languages and dietary patterns remains a challenge.

## Cultural, demographic and socio-economic considerations

No diet quality index is currently universally valid across all populations. For example, tools developed for Western adults may misclassify dietary adequacy in children, older adults or ethnic minority groups.

The UK Diet Quality Questionnaire showed how dietary patterns that represented higher and lower quality diets were strongly patterned by socio-economic status, with lower scores in more deprived areas^(10)^. This reflects the well-understood social patterning of Western diets in the UK population but may miss variation at local population level driven by cultural or ethnic dietary patterns. In addition, it was validated for use in adult populations, so is not generalisable to children.

The Global Diet Quality Questionnaire offers a modular, culturally flexible approach and has been tested in more than ninety countries^(25)^. It categorises foods into twenty-nine globally relevant groups and produces both nutrient-focused and food-based scores. While still undergoing validation, the Global Diet Quality Questionnaire is a promising tool for cross-cultural surveillance and research.

## Dietary pattern analysis as a complementary approach

A posteriori methods such as principal component analysis cluster analysis and reduced rank regression enable researchers to identify empirically derived dietary patterns^(26,27)^. These data-driven methods reveal how people actually eat, offering real-world insight that can inform tool development^(28)^.

Principal component analysis has been conducted in UK National Diet and Nutrition Survey data to define dietary patterns in adults which were associated with both nutrient intake, biomarkers, sociodemographic variables, BMI and smoking status^(9)^. It has been conducted in several studies in the ALSPAC longitudinal dataset to identify and explore dietary patterns in pregnancy and associations with infant and child outcomes^(29,30)^. Similarly, reduced rank regression has been used in case-control studies to identify patterns predictive of inflammation, adiposity, and insulin resistance^(31)^. These methods are particularly valuable for exploring novel dietary behaviours (e.g. high ultra-processed food intake) and for validating or refining existing indices.

However, dietary pattern analysis is resource-intensive and requires detailed, harmonised intake data. While not suitable for routine surveillance, these methods can guide the development of brief screeners and culturally specific tools.

## Conclusion and future directions

Measuring diet quality is critical for understanding dietary determinants of health, guiding public policy and monitoring dietary trends. However, current approaches are constrained by cultural variability, methodological limitations and a lack of harmonisation.

Future efforts should focus on:

- Developing and validating tools that combine food-based simplicity with nutrient-based rigour.
- Expanding validation studies to include children, ethnic minorities and low-income groups.
- Building flexible, globally adaptable instruments like the Global DQQ.
- Using dietary pattern analysis to refine tools and capture emerging behaviours.

Cross-country studies like European Prospective Investigation into Cancer and Nutrition and tools with universally applicable elements like the DQI offer valuable models for calibration and harmonisation. Integrating data from multiple sources – FFQ08, recalls, biomarkers and patterns can create a more nuanced picture of diet quality and support international efforts to reduce diet-related disease.
